# Dipeptidyl Peptidase-4 Is a Target Protein of Epigallocatechin-3-Gallate

**DOI:** 10.1155/2020/5370759

**Published:** 2020-02-11

**Authors:** Huimin Hou, Ying Wang, Chunshi Li, Jian Wang, Yanli Cao

**Affiliations:** ^1^Department of Endocrinology and Metabolism, Institute of Endocrinology, Liaoning Provincial Key Laboratory of Endocrine Diseases, The First Affiliated Hospital of China Medical University, China Medical University, Shenyang, Liaoning, China; ^2^Key Laboratory of Structure-Based Drug Design & Discovery, Ministry of Education, Shenyang Pharmaceutical University, Shenyang, Liaoning, China

## Abstract

Epigallocatechin-3-gallate (EGCG), a major active ingredient in green tea, has various health benefits. It affects glucose metabolism, but the mechanism is not well understood. This study aimed to identify targets of EGCG related to glucose metabolism. The core fragment of EGCG is a flavonoid. The flavonoid scaffold was used as a substructure to find proteins cocrystallized with flavonoids in the Protein Data Bank. The proteins identified were screened in PubMed for known relationships with diabetes. Dipeptidyl peptidase-4 (DPP4; PDB 5J3J) was identified following this approach. By molecular docking, the interactions of EGCG and DPP4 were assessed. To test the stability of the interactions between EGCG and DPP4, molecular dynamics simulation for 100 ns was performed using Desmond software. In vitro, the concentration of EGCG required to inhibit DPP4 activity by 50% (the IC50 value) was 28.42 *μ*M. These data provide a theoretical basis for intervention in glucose metabolism with EGCG.

## 1. Introduction

The occurrence of diabetes and impaired glucose tolerance is increasing globally [1]. In 2013, among adults in China, the prevalence of diabetes was 10.9% and that of prediabetes was 35.7% [2]. Studies of diabetes patients in China report low awareness rates of the diagnosis and poor treatment rates and glycemic control [2]. Current medications for type 2 diabetes mellitus are limited in that they are always associated with adverse effects such as hypoglycemia, weight gain, or bone loss, among others.

Tea is a cheap and commonly consumed human beverage, often considered a healthy habit [3, 4]. Green tea has important effects on human health, mainly attributed to its flavonoid-like polyphenols, such as catechins. Catechins included epigallocatechin gallate (EGCG), epigallocatechin (EGC), epicatechin gallate (ECG), epicatechin (EC), and catechin (C). EGCG is the major catechin in green tea extract [5]. Compounds in tea can reduce the risk of chronic illnesses including cardiovascular disease, cancer, arthritis, and diabetes [3–8]. However, the mechanism of the effect of EGCG on glucose metabolism is not well understood.

The enzyme dipeptidyl peptidase-4 (DPP4) is present in many human tissues, including the pancreas, liver, and adipose cells. As a cell-surface protease, DPP4 selectively cleaves an N-terminal dipeptide from peptides in which the penultimate amino acid is proline or alanine (for example, glucose-dependent insulinotropic polypeptide (GIP) and glucagon-like peptide-1 (GLP-1)), inactivating them [9, 10]. GLP-1 is secreted from L-cells of the intestine. Binding of GLP-1 to G-protein-coupled receptors on the surface of beta cells leads to an increase in intracellular cAMP, activation of Epac1 and 2, and finally insulin release [11]. Accordingly, DPP4 inhibitors have clinical benefits in patients with diabetes mellitus [12]. A great number of DPP4 inhibitors with novel structures and high activity and selectivity have been reported, which can be divided into three categories: peptidomimetics, peptides, and nonpeptidomimetic compounds; many of them have been designed based upon the inhibitory potency, absorption, oral bioavailability, selectivity, and half-life [13]. In addition to blood glucose regulation, DPP4 inhibitors may also have extra-glycemic effects such as myocardial protection, lowering blood pressure, reducing the expression of local inflammatory factors, and improving vascular endothelial function [14]. However, side effects such as hemolysis, angioedema, and rheumatoid arthritis have also been observed [13]. Therefore, there is interest in dietary therapies for the prevention and treatment of diabetes without adverse reactions. Synthesis of important chemical entities and some computer-based research methods such as virtual screening, molecular docking, and molecular dynamics simulation offer a new strategy for the discovery and design of novel inhibitors [15, 16].

The aim of this study was to identify novel target proteins of EGCG that are relevant to diabetes and thus provide new data on the relationship between EGCG and glucose metabolism.

## 2. Materials and Methods

### 2.1. Target Protein Acquisition by Ligand Search

#### 2.1.1. EGCG as a Search Term in Database

Initially, the Protein Data Bank (PDB, http://www.rcsb.org/pdb/) was searched for crystal structures with EGCG as a cocrystallized ligand. Five structures were found—PDB codes 1JNQ [17], 2KDH [18], 3NG5 [19], 3OOB [20], and 4AWM [21]. None of these proteins is diabetes-related. To expand the range of proteins of interest, a substructure search was used.

#### 2.1.2. Substructure Search

**Figure 1:**
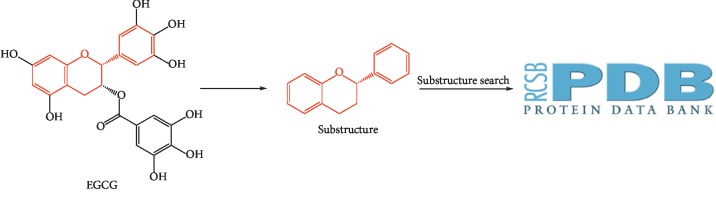
Graphical summary of substructure search.

The EGCG flavonoid scaffold (the red part in Figure 1) was taken as a substructure of interest, drawn in Chembiodraw Ultra 14.0, and processed using SMILES, and the PDB was searched for proteins cocrystallized with this structure. Proteins obtained in the above way were retrieved in the PubMed database to find out which protein plays an important role in glucose metabolism, and then molecular docking between the target protein and EGCG was performed.

### 2.2. Molecular Docking

#### 2.2.1. Protein Preparation

X-ray crystal structures of proteins of interest were downloaded from the PDB. The DPP4 protein structure was prepared using Schrödinger's Protein Preparation Wizard in Maestro v9.7 [22]. Crystallographic water molecules were deleted, protein structure defects were corrected, and missing side-chain atoms and hydrogen atoms were added. Acidic and basic amino acid side chains were placed in the expected ionization state at pH 7.4.

#### 2.2.2. Ligand Preparation

The chemical structure of EGCG was prepared in Chembiodraw (Ultra 14.0), converted into a 3D structure, saved as a mole file, imported into the Maestro Project Table in Schrödinger, and optimized using the OPLS-2005 force field in the LigPrep module of the software suite [23]. Hydrogen atoms were added and the hydrogen bonds were optimized.

#### 2.2.3. Molecular Docking

Molecular docking is a method that predicts the preferred orientation of one molecule to a second when bound to each other to form a stable complex. In addition, this method could also be used to predict the affinity of a ligand within the binding pocket of the target of interest [24–27]. Here, molecular docking simulations were performed using Glide 9.7 (version 2014) in Schrödinger [28].

A grid box was generated around the DPP4 active site (see Section 2.2.1). The active site was defined as a 10 Å radius around the ligand. EGCG was docked into the active site of the target protein using extra precision mode in Glide without any constraints and default settings for all other parameters. Docking calculations were based on the Glide Score.

### 2.3. Molecular Dynamics (MD) Simulation

Nowadays, diverse techniques for MD constitute important computational tools to study protein drug targets and protein interactions with ligands at the molecular level, particularly for examining the motion of individual atoms, which can be tracked over time. Thus, to verify the accuracy of the docking results for EGCG and DPP4 and to further explore the specific molecular interactions of DPP4 with EGCG, MD simulation was performed.

Water molecules were added using Desmond and then 0.15 mol/L NaCl was added to make the whole system isotonic with the human body fluid environment. Counter ions were added to make the simulation system electrically neutral. The simulation was performed in NPT ensemble class with the temperature fixed at 300 k using Nose-Hoover thermostat and pressure at 1.01325 bar using Martyna-Tobias-Klein barostats. The MD simulation of the complex of DPP4 with EGCG was carried out for 100 ns.

### 2.4. DPP4 Protease Inhibition Assay

To evaluate the inhibitory activity on DPP4, the DPPIV-Glo™ Protease Assay (Promega, USA) was used to assess the effect of EGCG on DPP4 enzyme activity in vitro. At the same time, the inhibitory effect of other compounds present in tea such as epigallocatechin, epicatechin gallate, epicatechin, and catechin on DPP4 activity was also determined. DPP4 enzyme and EGCG were purchased from Sigma. Sitagliptin (Sigma, USA), a DPP4 inhibitor [29], was used as a reference compound. Epigallocatechin, epicatechin gallate, epicatechin, and catechin were obtained from bidepharm (Shanghai, China). Half maximal inhibitory concentration (IC50) values were determined by nonlinear regression in GraphPad Prism 6 software.

## 3. Results

### 3.1. Substructure Search

**Figure 2:**
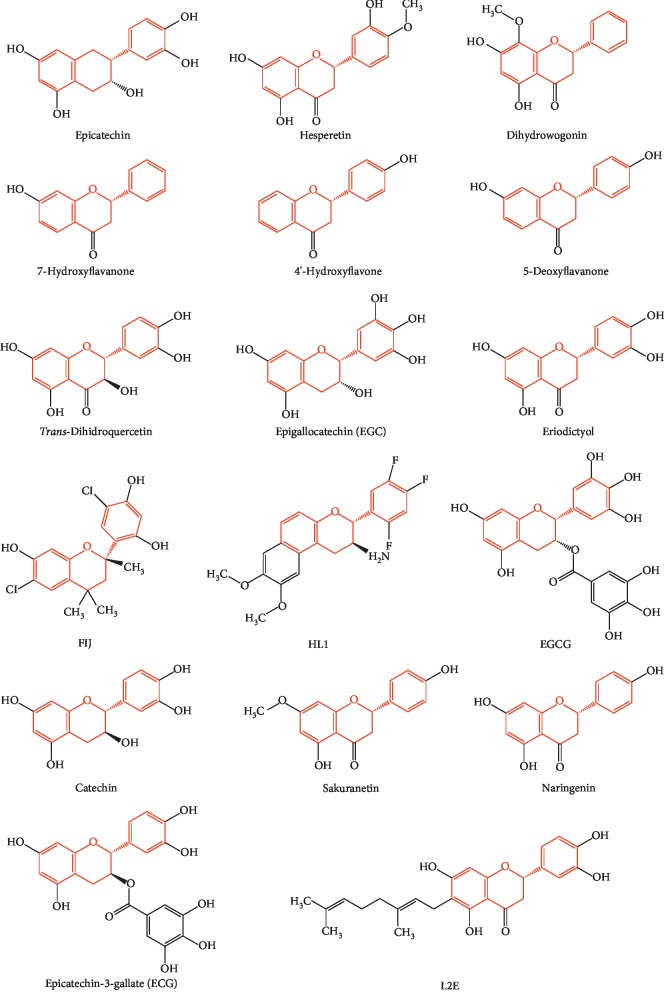
Chemical structures of ligands containing the flavonoid scaffold identified by substructure search.

EGCG was searched in the PDB database as a search term, four structures cocrystallized with EGCG and one structure cocrystallized with EGC were acquired, and none of them was related to glucose metabolism. In order to obtain proteins that may bind to the flavonoid scaffold, substructure search was used. Seventeen chemical structures of ligands containing the flavonoid scaffold were identified by substructure search (Figure 2), and 40 cocrystal structures of proteins with the flavonoid scaffold substructure of EGCG were identified in the PDB (PDB IDs 6MS8, 6G8H, 6AWT, 6AWV, 6AX0, 6DHL, 6F6A, 5WKR, 5WKS, 5J3J, 5F8P, 5JDC, 4MA6, 4D06, 4KZQ, 4C94, 4C9I, 4EH3, 4DEU, 4AWM, 4A87, 3NG5, 3TVQ, 3OOB, 3I52, 3D04, 2KDH, 2NNL, 2UXU, 2C29, 2BRT, 1GP5, 1JNQ, 1JX0, 1JX1, 1FM7, 1FM8, 1JEP, 1EYQ, and 1CGK); the information about these proteins is reported in Table 1. The relationship between these proteins and diabetes was investigated in PubMed. Human DPP4 (PDB ID 5J3J) was found to be associated with glucose metabolism, and various DPP4 inhibitors have been released as therapeutic drugs for type 2 diabetes. Since plenty of studies have shown that EGCG may have the potential to improve the glycemic profiles in patients with diabetes, we selected DPP4 protein as a receptor for molecular docking to study whether EGCG can imitate the action of DPP4 inhibitors with few side effects.

### 3.2. Molecular Docking

**Figure 3:**
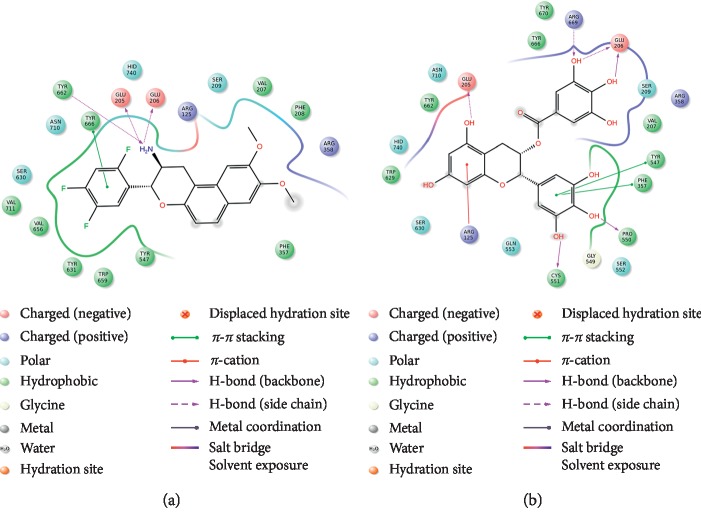
Interaction of the active site of dipeptidyl peptidase-4 (DPP4) with (a) HL1 and (b) epigallocatechin-3-gallate (EGCG).

Initially, we examined molecular docking between DPP4 and the ligand HL1, which were cocrystallized in PDB structure 5J3J. A molecular docking result is considered accurate if the root mean square deviation (RMSD) value is <2.0 Å [30]. DPP4 and HL1 were docked (Figure 3(a)). The RMSD value obtained using the Glide algorithm was 0.426 Å, so the docking result was considered accurate. The tetrahydro-2H-pyran-3-amine nitrogen atoms of HL1 form hydrogen bonds with the side chains of residues Glu205, Glu206, and Tyr662, and the 2, 4, 5-trifluorobenzene group provides *π*–*π* interactions with Tyr666.

EGCG was docked into the crystal structure of DPP4. In the molecular docking simulation, EGCG bound to DPP4 where HL1 binds, with a Glide Score of −9.439 kcal/mol for the most stable structure (Figure 3(b)). Hence, the affinity for EGCG was higher than that for HL1 (Glide Score: −5.628 kcal/mol). Active site residues Glu205, Glu206, Pro550, Cys551, and Arg669 formed hydrogen bonds with EGCG. Furthermore, Arg125 interacted through a *π*–cation interaction with the 5,7-dihydroxybenzene ring of EGCG, and the 3,4,5-trihydroxybenzene ring made *π*–*π* interactions with Phe357 and Tyr547. Thus, there were more interactions between DPP4 and EGCG than between DPP4 and HL1, possibly accounting for the tighter binding. These predicted interactions suggest that EGCG may be able to bind with DPP4.

### 3.3. MD Simulation

**Figure 4:**
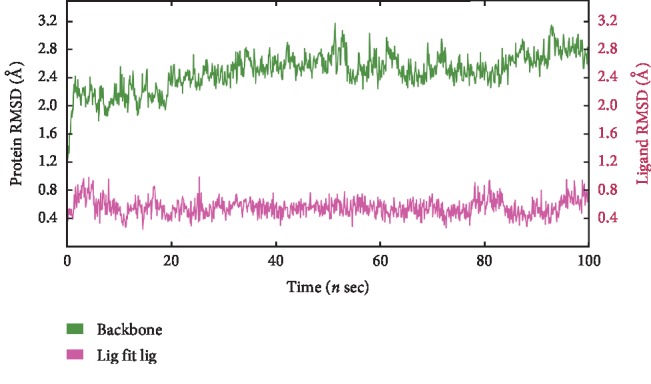
Root mean square deviations (RMSD) of backbone atoms of DPP4 and ligand EGCG during 100 ns molecular dynamics (MD) simulation.

The root mean square deviation (RMSD) is an indicator that describes the average change in displacement of an atom in a specific molecular conformation with respect to a reference conformation [31]. The RMSD value of the DPP4 protein backbone was initially 1.2 Å, which then increased and stabilized at 2.6 Å (Figure 4). The system was equilibrated.

Root mean square fluctuation (RMSF) refers to the root mean square displacement of each residue of a frame conformation relative to the average conformation, which is used to determine the flexibility of a region of the protein. RMSF can describe local changes along the protein chain, which were calculated throughout the simulation. In an RMSF plot, the peak indicates which region of the protein fluctuates most during the simulation, while lower RMSF values represent smaller conformational change. The RMSF values of the residues during the MD simulation of DPP4 binding EGCG are shown in [Supplementary-material supplementary-material-1]. The amino acid residues with the highest RMSF values interact with the ligand, such as Glu206, which forms hydrogen bonds with EGCG. Most residues fluctuated within 2.0 Å, and very few residues had an RMSF value >3.0 Å. As can be seen from the RMSD and RMSF plots, the protein and ligand did not show large fluctuations in the 100 ns MD simulation process, which indicated that the complex was stable during the simulation.

**Figure 5:**
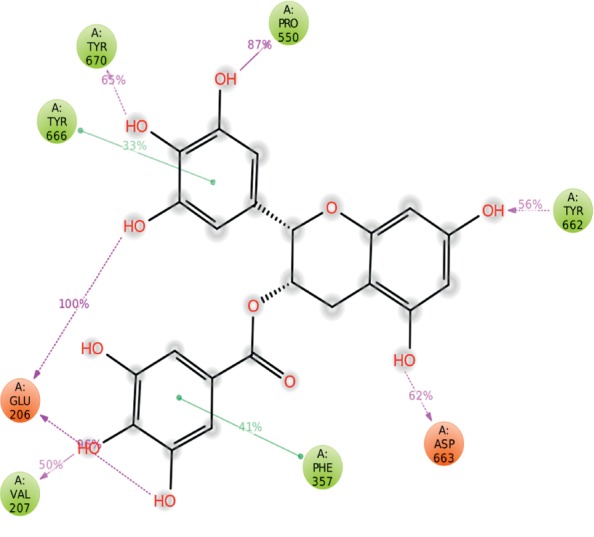
DPP4–EGCG interaction percentages during 100 ns MD simulation. Interactions that occurred for >30.0% of the simulation time are shown.

When the system was stable, interaction stability of the complex was monitored. In molecular docking analysis, the complex of EGCG and DPP4 showed six hydrogen bonds, one involving each of Glu205, Pro550, Cys551, and Arg669, and two involving Glu206. In MD simulation, Glu206 formed two hydrogen bonds with hydroxyl groups, for 96% and 100% of the total simulation time, respectively. Val207 formed a hydrogen bond with a hydroxyl group for 50% of the total simulation time, Pro550 with a hydroxyl group for 87%, Tyr662 with a hydroxyl group for 56%, Asp663 with a hydroxyl group for 62%, and Tyr670 with a hydroxyl group for 65% of the total simulation time. Phe357 and Tyr666 showed *π*–*π* interactions with the benzene rings for 41% and 33% of the total simulation time, respectively (Figures 5 and [Supplementary-material supplementary-material-1]).

Protein–ligand interactions include hydrogen bonds, hydrophobic and ionic interactions, and water bridges. A simulation interaction diagram showed that hydrogen bonds played an important role in the binding of EGCG within the active site of DPP4. Hydrophobic interactions also accounted for a large part of the binding. Stacked bar charts were normalized over the course of the trajectory ([Supplementary-material supplementary-material-1]). Values >1.0 were possible as some protein residues made multiple contacts of the same subtype with the ligand. EGCG interacted with residue Glu206 throughout the course of the dynamic simulation. Other residues that showed prominent interactions with EGCG were Val207, Phe357, Pro550, Tyr662, Asp663, Tyr666, and Tyr670. Interactions through hydrogen bonds between Glu206 of DPP4 and EGCG were maintained effectively for 196% of the simulation time, which was reasonable as Glu206 formed more than one contact with EGCG.

### 3.4. DPP4 Protease Inhibition Assay

**Figure 6:**
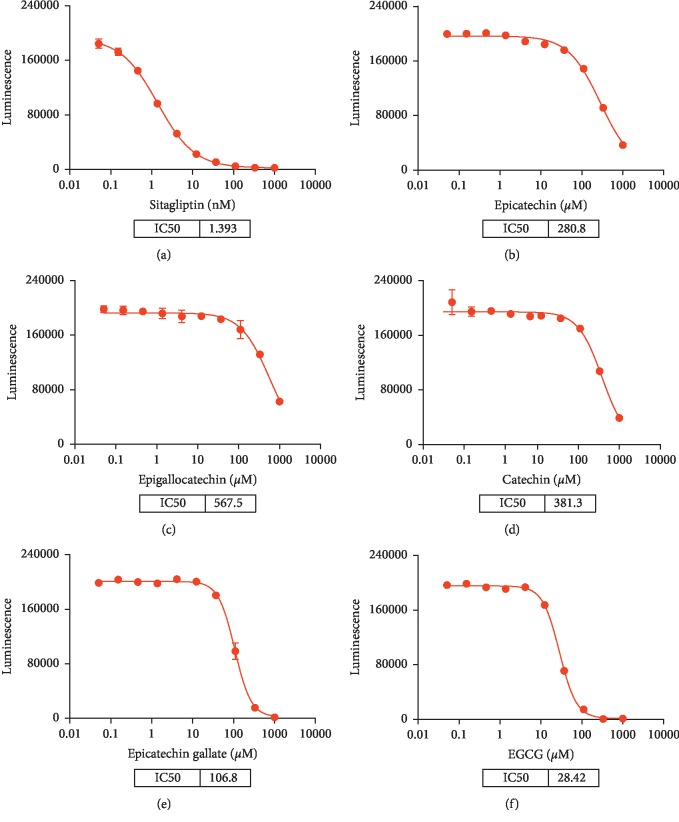
Activity of DPP4 in DPPIV-Glo™ Protease Assay in the presence of (a) sitagliptin, (b) epicatechin, (c) epigallocatechin, (d) catechin, (e) epicatechin gallate, and (f) EGCG.

The data showed that sitagliptin could potentially inhibit DPP4 activity with an IC50 value of 1.393 nM. Among the five catechins, EGCG exhibited the powerful inhibition of DPP4 activity with IC50 = 28.42 *μ*M (Figure 6). All other compounds including epicatechin (IC50 = 280.8 *μ*M), epigallocatechin (IC50 = 567.5 *μ*M), catechin (IC50 = 381.3 *μ*M), and epicatechin gallate (IC50 = 106.8 *μ*M) were less active than EGCG.

## 4. Discussion

Consumption of tea has many beneficial health effects, such as alleviation of metabolic syndrome, prevention of obesity, and protection against type 2 diabetes [32, 33], but some specific mechanisms have yet to be elucidated. In this study, we aimed to find diabetes-related targets of EGCG, a major component of tea polyphenols. By flavonoid scaffold substructure search, we found that DPP4, a serine protease, may be a potential target of EGCG. Molecular interaction of DPP4 and EGCG was studied by performing molecular docking and MD simulation. Molecular docking showed that EGCG interacted with residues Arg125, Glu205, Glu206, Phe357, Tyr547, Pro550, Cys551, and Arg669. MD simulation analyzed the interaction of the ligand–protein (EGCG–DPP4) complex in physiological conditions and evaluated the stability of the complex over time, which showed additional hydrogen bonds and *π*–*π* stacking on different timescales. Inhibitory activity of EGCG toward DPP4 was confirmed using the DPPIV-Glo™ Protease Assay. At the same time, the effects of other catechins on DPP4 in tea had also been evaluated obtaining catechin, epicatechin, epigallocatechin, and epicatechin gallate.

As far as we know, this is the first study on the effect of EGCG on DPP4 enzyme activity. A previous research predicted that epicatechin derivate may have an inhibitory effect on DPP4 activity through a virtual screening method [34], which is consistent with our findings. A study [35] reported the docking results of EGCG with DPP4 but not MD simulation or activity assay in vitro. We further explored the mechanisms of the tea polyphenol EGCG that exerts antidiabetic effects using different docking software and additional MD simulations that are closer to physiological conditions and activity assays. Our novel data have shown that EGCG has the potential for the treatment of diabetes.

DPP4 processes the peptide GLP-1, which ultimately influences insulin release. The gliptin family of drugs that inhibit DPP4 has attracted interest in treating patients with type 2 diabetes. Although currently available DPP4 inhibitors have good safety and tolerability [36], and most patients have only mild adverse reactions, there is interest in dietary therapies for diabetes that have relatively low efficacy but no adverse reactions. Our results suggest that EGCG and DPP4 may interact, which could provide a theoretical basis for improving diabetes-related metabolic abnormalities by tea polyphenols.

DPP4 inhibitors are therapeutic drugs for diabetes with anti-inflammatory effects. One study showed that oral administration of sitagliptin not only reduced the level of HbA1c in diabetic patients but also reduced the levels of various proinflammatory cytokines such as IL-6, C-reactive protein, free fatty acids, and TLR4 [37]. Anti-inflammatory effects of EGCG have also been demonstrated [38]. Our study shows that EGCG inhibits DPP4, which may be a mechanism of the anti-inflammatory effects of EGCG.

Our research provides new ideas about using polyphenols to improve blood glucose metabolism. There are a few attractive points that are not addressed in this article. It is important to have positive and negative control for MD simulation. It can give insight on the binding interaction of EGCG with DPP4. It would also be interesting to study the effect of EGCG on the DPP4 family such as DPP8 and DPP9. In addition, the relationship between DPP4 and EGCG merits further in vivo exploration.

## Figures and Tables

**Table 1:** Description of 40 proteins cocrystallized with the flavonoid scaffold.

PDB ID	Name	Uniprot ID	Protein length	Organism
6MS8	Chalcone-flavonone isomerase family protein	B7FJK3	224	*Medicago truncatula*
6G8H	TetR/AcrR family transcriptional regulator	A0A2A6N3G4	214	*Bradyrhizobium diazoefficiens*
6AWT, 6AWV,				
6AX0, 4MA6	Ara h 8 allergen	Q6VT83	157	*Arachis hypogaea*
6DHL	Glutamate dehydrogenase 1	P00366	496	*Bos taurus*
6F6A	Glutathione transferase	A0A384E145	246	*Trametes versicolor*
5WKR, 5WKS	Engineered chalcone isomerase	N/A	223	*N/A*
5J3J	Dipeptidyl peptidase-4	P27487	734	*Homo sapiens*
5F8P	Alpha-ketoglutarate-dependent dioxygenase FTO	Q9C0B1	476	*Homo sapiens*
5JDC	Pteridine reductase	O76290	288	*Trypanosoma brucei brucei*
4D06	Chalcone isomerase	V9P0A9	283	*Eubacterium ramulus*
4KZQ	Tankyrase-2	Q9H2K2	191	*Homo sapiens*
4C94	Pathogenesis-related 10 (Pr-10) Fra a 3	D0E0C7	161	*Fragaria ananassa*
4C9I	Pathogenesis-related 10 (Pr-10) Fra a 1E	Q256S2	162	*Fragaria ananassa*
4EH3	Mitogen-activated protein kinase 14	Q16539	360	*Homo sapiens*
4DEU, 3NG5	Transthyretin	P02766	147	*Homo sapiens*
4AWM	Polymerase acidic protein	C3W5S0	192	*Influenza A virus*
4A87	Major pollen allergen bet V 1-A	P15494	159	*Betula pendula*
3TVQ	Multifunctional cyclase-dehydratase-3-O-methyl transferase tcmN	P16559	169	*Streptomyces glaucescens*
3OOB	Peptidyl-prolyl cis-trans isomerase NIMA-interacting 1	Q13526	163	*Homo sapiens*
3I52	Putative leucoanthocyanidin reductase 1	Q4W2K4	346	*Vitis vinifera*
3D04	(3R)-hydroxymyristoyl-acyl carrier protein dehydratase	Q5G940	159	*Helicobacter pylori*
2KDH	Troponin C	P63316	72	*Homo sapiens*
2NNL	Dihydroflavonol 4-reductase	P51110	337	*Vitis vinifera*
2UXU	HTH-type transcriptional regulator TtgR	Q9AIU0	210	*Pseudomonas putida*
2C29	Dihydroflavonol 4-reductase	P93799	337	*Vitis vinifera*
2BRT, 1GP5	Leucoanthocyanidin dioxygenase	Q96323	356	*Arabidopsis thaliana*
1JNQ	Lipoxygenase-3	P09186	857	*Glycine max*
1JX0, 1JX1				
1FM7, 1FM8	Chalcone-flavonone isomerase 1	P28012	222	*Medicago sativa*
1JEP, 1EYQ				
1CGK	Chalcone synthase	P30074	389	*Medicago sativa*

## Data Availability

The data used to support the findings of this study are included within the article.

## Abbreviations

DPP4: Dipeptidyl peptidase-4
EGCG: Epigallocatechin-3-gallate
IC50: Half maximal inhibitory concentration
PDB: Protein data bank.

## Supplementary Materials

Supplementary Materials Figure S1: root mean square fluctuation (RMSF) of the backbone of DPP4 during 100 ns MD simulation. Figure S2: a representation of the interactions and contacts over time. The top panel shows the total number of specific contacts DPP4 makes with EGCG over the simulation. The bottom panel shows which residues interact with EGCG in each trajectory frame. Figure S3: normalized stacked bar chart representation of interactions and contacts over the course of the MD simulation trajectory.
