# Supplementary material for: Dipeptidyl Peptidase-4 Is a Target Protein of Epigallocatechin-3-Gallate
Source: Biomed Res Int. 2020 Feb 11;2020:5370759. doi: 10.1155/2020/5370759 (PMC7036097; doi:10.1155/2020/5370759)
Supplement: Supplementary Materials — Figure S1: root mean square fluctuation (RMSF) of the backbone of DPP4 during 100 ns MD simulation. Figure S2: a representation of the interactions and contacts over time. The top panel shows the total number of specific contacts DPP4 makes with EGCG over the simulation. The bottom panel shows which residues interact with EGCG in each trajectory frame. Figure S3: normalized stacked bar chart representation of interactions and contacts over the course of the MD simulation trajectory. [file 5370759.f1.pdf]

Supplementary materials

Figure S1. Root mean square fluctuation (RMSF) of the backbone of DPP4 during 100-ns MD simulation.

Figure S2. A representation of the interactions and contacts over time. The top panel shows the total number of specific contacts DPP4 makes with EGCG over the simulation. The bottom panel shows which residues interact with EGCG in each trajectory frame.

Figure S3. Normalized stacked bar chart representation of interactions and contacts over the course of the MD simulation trajectory.

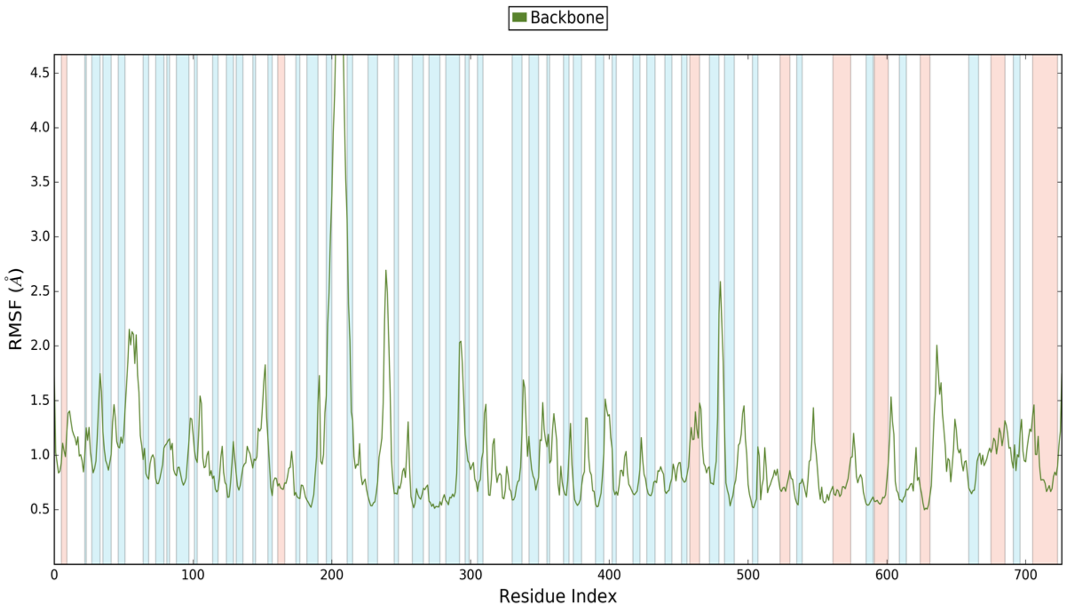

Figure S1

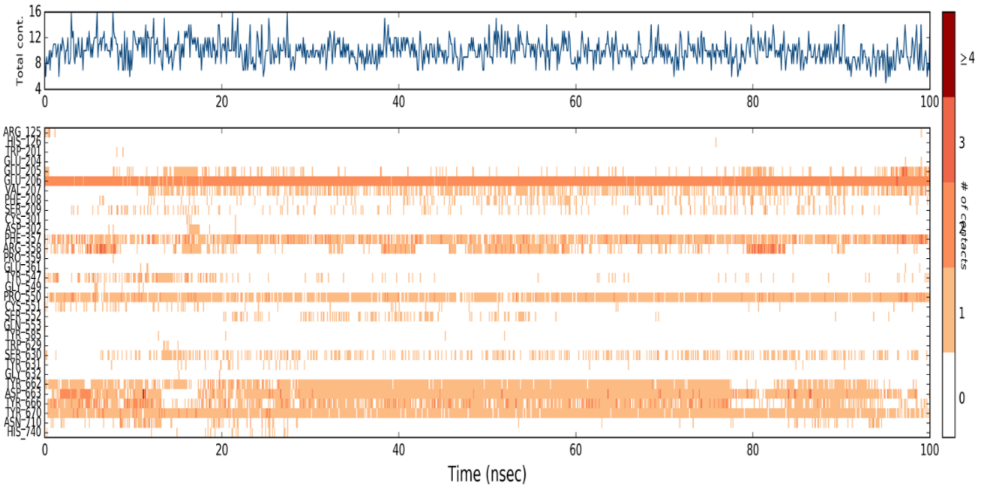

Figure S2

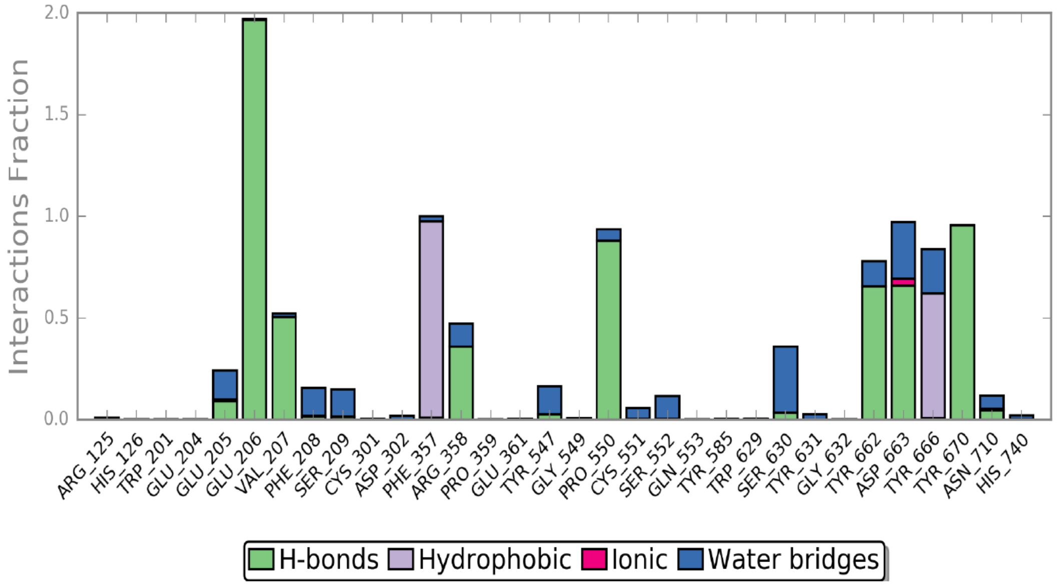

Figure S3
